# Supplementary material for: Antifungal potential of Rhinacanthus nasutus extracts against the pathogenic fungus Cryptococcus neoformans
Source: PLoS One. 2025 Dec 19;20(12):e0339279. doi: 10.1371/journal.pone.0339279 (PMC12716790; doi:10.1371/journal.pone.0339279)
Supplement: Attachment — Submitted filename: Point-by-Point Rebuttal .pdf [file pone.0339279.s007.pdf]

Point-by-Point Rebuttal to Editorial Decision Letter

Manuscript Title: Antifungal potential of *Rhinacanthus nasutus* extracts against pathogenic fungi including *Cryptococcus neoformans* and *Candida auris*

We would like to thank the editorial team for the comments and the opportunity to revise and resubmit our manuscript. Below, we address the concerns raised in the editorial letter, point by point:

Editorial Comment 1:

"We would expect to see the full name of the farm from which you obtained the plant, or its specific location."

Response:
Thank you for the feedback. We have now included the full name and specific location of the farm from which the plant was obtained in the Materials and Methods section. The revised text reads:
" Leaves and roots of R. nasutus were collected between February to July 2024 from the Rammasak Women's Herbal Group Farm, located in Rammasak Subdistrict, Ang Thong Province, Thailand (14.6954°N, 100.2719°E)."

Editorial Comment 2:

"Additionally, we require the authentication of the plant used."

Response:
We appreciate this important point. We have now included authentication details. The plant was authenticated by Prof. Dr. Paweena Traiperm, a specialist in plant taxonomy from the Department of Plant Science, Faculty of Science, Mahidol University. The authentication statement has been added to the revised Methods section.

Editorial Comment 3:

"Experiments and analyses must be conducted rigorously, with appropriate controls; methods must be described in sufficient detail for others to replicate the analyses; and conclusions must be supported by the results presented."

Response:
We acknowledge the importance of methodological clarity and scientific rigor. In the revised manuscript. We have revised the Methods section to include full details on sample preparation, experimental controls. Additional data are included in the Supplementary Materials to support

transparency and reproducibility. We also have reviewed the Results and Discussion sections to ensure all conclusions are clearly linked to the data presented.

We respectfully request that the editorial office reconsider our revised manuscript for publication in PLOS ONE. A revised version of the manuscript and a cover letter detailing the resubmission will be provided as per journal guidelines.

Sincerely,

Dr. Sittinan Chanarat
On behalf of all co-authors
