# Supplementary material for: Antifungal potential of Rhinacanthus nasutus extracts against the pathogenic fungus Cryptococcus neoformans
Source: PLoS One. 2025 Dec 19;20(12):e0339279. doi: 10.1371/journal.pone.0339279 (PMC12716790; doi:10.1371/journal.pone.0339279)
Supplement: Attachment — Submitted filename: Point-by-point response 250916.pdf [file pone.0339279.s008.pdf]

Point-by-point response

Reviewer #1:

The authors analyzed the effects of the antifungal activity of R. nasutus extracts and their impact on the virulence factors of Cryptococcus neoformans. The subject is adequate with the journal's scope. The argument to support the research is valid, the manuscript is well written and understandable to a specialist readership, and organization, and the article's structure is good and in agreement with the journal instructions for authors. The quality of the article needs to be improved. With editing and some revisions, I feel that this manuscript will be suitable for publication.

Response: We thank the reviewer for the positive assessment of our manuscript. In accordance with the suggestion, we have carefully revised and edited the text to improve clarity, readability, and overall quality.

1. Abstract and introduction are well written however, the methodology should be improved for example, the methods for antifungal testing using spot assay, it is not clear that the antifungal effect of the solvent (25% glycerol in ethanol) was evaluated

Response: We have thoroughly revised the Methods section to improve clarity. In particular, the effect of the solvent was evaluated in every experiment, as indicated by the inclusion of a “solvent” control. Additional details have also been added to each experiment to enhance transparency and readability. We appreciate the reviewer’s constructive suggestions, which helped us strengthen the manuscript.

2. Also, it is very important to mention the statistical analysis used to interpret the results

Response: We apologize for the earlier omission and have now included the statistical analysis section under Materials and Methods. We thank the reviewer for this constructive suggestion, which has improved the clarity of our manuscript.

3. For the results section, the paragraph stating the preparation of and HPLC analysis of Rhinacanthus nasutus extracts (lines 159 to 170) should be moved to methodology section

Response: We thank the reviewer for the suggestion and have rewritten the manuscript accordingly.

*4. The figure legend of figure 3 should include a clarifying sentence about the p value and the meaning of the Asterix (ns,**, ***)*

Response: We apologize for the earlier omission and have now added the statistical analysis and P-value in this figure legend.

Reviewer #2:

This manuscript explores the antifungal activity of R. nasutus extracts against key fungal pathogens and focuses especially on Cryptococcus neoformans. The findings regarding the inhibition of virulence factors such as melanin production and capsule formation are promising and may support the development of plant-based antifungal therapeutics. However, the study suffers from multiple critical limitations in design, scope alignment, and scientific rigor, which must be addressed before consideration for publication.

While the study title highlights the antifungal potential of Rhinacanthus nasutus extracts against pathogenic fungi, including Cryptococcus neoformans and Candida auris, the introduction and overall narrative focus almost exclusively on C. neoformans. Other listed pathogens, particularly Candida auris, are barely mentioned or contextualized. While the title suggests a broad antifungal evaluation including Candida auris, the experimental work and in-depth analyses are almost entirely limited to C. neoformans.

Response: We thank the reviewer for pointing this out and agree that the manuscript may not provide sufficiently convincing data regarding *C. auris*. In the revised version, we have removed all data related to *C. auris* and modified the title accordingly, without altering the main concept or overall content of the manuscript. The revised title now is: *"Antifungal potential of Rhinacanthus nasutus extracts against the pathogenic fungus Cryptococcus neoformans."*

Why was 25% glycerol in ethanol selected in extraction method? Was this optimized for antifungal activity, stability, or solubility? All extracts used 5 g/20 mL, which is a good control measure, but it's unclear whether the extract concentrations were normalized for rhinacanthin-C content before use in assays.

Response: The extraction solvent of 25% glycerol in ethanol was selected because it has been shown that glycerol enhances the solubility and stability of rhinacanthin-C, the principal antifungal compound in *R. nasutus*, while ethanol acts as a safe organic solvent that is also compatible with topical formulations (1). This combination enabled efficient extraction and improved shelf stability of the extracts. As rhinacanthin-C is not commercially available and we are unable to synthesize it, we could not normalize the extracts based on its exact content. Instead, concentrations used in our assays are expressed as the dry weight of plant material per solvent volume or final volume used in each assay.

- why was the concentration of 8mg/ml used in Fig.2A and exp onwards.

Response: We titrated and evaluated the effects in growth assays, and found that 8 mg/mL (calculated as dry weight per final volume) was the most effective concentration for suppressing yeast growth (Supplementary Fig. S1). Therefore, we selected this concentration for subsequent experiments.

What was used as a standard for rhinacanthin-C quantification? Was a calibration curve generated using a purified standard? The actual rhinacanthin-C content should be reported, even if briefly, in this section or in results. This supports the reproducibility and pharmacological relevance of the extract.

Response: As stated in the revised manuscript, because a purified rhinacanthin-C standard is not commercially available and we were unable to synthesize it chemically, we could not perform direct quantification in this study. Instead, we referred to previously published work that used the same extraction approach and, based on that, we estimated the approximate concentration of rhinacanthin-C in our extract. This rough estimation has now been included in the Results section to provide context for reproducibility and pharmacological relevance.

In antifungal Spot Assay Clarify how the dilution of the extracts affected final concentrations on the plates. Were technical replicates or biological replicates included? If so, how many?

Response: We apologize for the earlier ambiguity regarding extract concentrations. All concentrations are expressed as the dry weight of plant material relative to the solvent or final assay volume, and this has now been clarified explicitly in both the *Materials and Methods* and in the relevant experimental descriptions. In addition, all experiments in this study were performed in at least duplicate, and this information has been added to the figure legends for clarity.

The use of the FIC index is appropriate and follows standard protocols. You should specify the concentration range used for each drug and extract. Consider adding how visual endpoints (growth/no growth) were defined, and whether a reference drug-only control was included in the plate layout.

Response: We apologize for the mistaken labeling in Figure 2B–2D. In accordance with standard protocols, the bottom-left panel in each figure represents the drug-free control, while the bottom row shows the effect of *R. nasutus* root extract alone. The figure has been corrected accordingly. We have also added the concentration ranges for each antifungal drug and the *R. nasutus* root extract, along with the endpoint criteria used to define the minimum inhibitory concentration (MIC), in the Materials and Methods section.

You may clarify whether the melanin pigmentation was quantified, or only qualitatively assessed.

Response: In the original manuscript, melanin pigmentation was not quantified. In the revised version, we have included quantitative analysis using the method described in our in-press paper (2). The quantification procedure is now detailed in the *Materials and Methods* section.

Specify whether microscopy was used for cell and capsule measurements and what stains (e.g., India ink) or imaging methods were applied.

Response: We used standard microscopy and capsule staining by the India ink method. All quantifications with ImageJ were performed following previously published protocols, which are now cited and described in greater detail in the Materials and Methods. We thank the reviewer for this constructive suggestion.

Indicate the number of fields or cells counted for morphological assessments.

Response: All measurements and statistical analyses were based on n = 50 cells, which is now stated clearly in both the Materials and Methods section and the figure legends.

Claims about inhibition of capsule size, melanin production, and morphological changes are made without clear statistical analysis (e.g., p-values, error bars, replication count).

Response: We apologize for the missing information. In the revised manuscript, we have added statistical details, including *p*-values and replication counts/*n* (for cells counted). We thank the reviewer for this helpful suggestion, which has improved the clarity and rigor of our manuscript.

One major limitation of the discussion is the complete absence of citations to previous literature, particularly in interpreting findings or situating them within the broader scientific context. For a study investigating the antifungal effects of Rhinacanthus nasutus, this omission is problematic for several reasons: No comparison with previous antifungal studies: Although prior reports were cited in the Introduction regarding R. nasutus's antifungal, antioxidant, and antibacterial properties, the Discussion does not refer back to those or any other comparative studies. This makes it unclear whether the observed effects (e.g., melanin suppression, capsule reduction, or synergy with antifungals) are novel, consistent with previous findings, or contradictory.

Response: We thank the reviewer for highlighting this important point. In the revised manuscript, we have substantially revised the Discussion to address this limitation. We now cite relevant literature on the antifungal, antioxidant, and antibacterial properties of *Rhinacanthus nasutus*, as

well as studies on its bioactive compound rhinacanthin-C. In addition, we have incorporated comparisons with previous antifungal studies to contextualize our findings on melanin suppression, capsule reduction, and synergistic interactions with antifungal drugs. These revisions ensure that our observations are clearly situated within the broader scientific context and allow readers to appreciate how our results align with or extend existing knowledge.

No contextualization of synergy data: The authors report near-synergistic interactions with antifungal drugs, but fail to cite similar studies that have examined combinations of plant extracts with standard antifungals—particularly against Cryptococcus neoformans or Candida auris.
Response: We appreciate the reviewer's suggestion. In the revised manuscript, we have expanded the Discussion to include relevant studies on the interactions between plant extracts and standard antifungal agents — particularly those targeting *C. neoformans* — and have added the appropriate references.

No references for virulence factor relevance: The discussion describes melanin and capsule reduction as important outcomes, which is accurate, but no references are provided to support the importance of these virulence traits in C. neoformans pathogenicity.
Response: We thank the reviewer for this constructive suggestion. In the revised manuscript, we have added appropriate references to support the importance of capsule formation and melanin production as key virulence factors in *C. neoformans* pathogenicity.

No discussion of rhinacanthin-C evidence: The authors suggest rhinacanthin-C may be an active compound based on HPLC data, but do not cite any literature linking rhinacanthin-C to antifungal activity, leaving this claim unsubstantiated.
Response: We appreciate this valuable comment and have substantially revised the Discussion section of the manuscript accordingly. We thank the reviewer for this helpful suggestion.

The decision to focus solely on C. neoformans, based on "pronounced potency," is understandable, but should not result in complete omission of C. auris, especially when this pathogen is highlighted in the title and introduction. Consider extending virulence or synergy assays to C. auris, or explicitly explain its exclusion in the methods and discussion. Replication details, error analysis, and visualization standards should be improved to enhance reproducibility.
Response: We thank the reviewer for this thoughtful comment. In the revised manuscript, we have addressed the concern by removing all references to *C. auris* from the title and the main

text, as our data on this pathogen were limited and not sufficiently convincing. We now focus exclusively on *C. neoformans*, for which we obtained robust and reproducible findings. In addition, we have revised the *Materials and Methods* and *Discussion* sections to explicitly explain the scope of the study and the rationale for focusing on *C. neoformans*.

Regarding reproducibility, we have clarified replication details and added explicit statements on biological replicates in the *Materials and Methods* and figure legends. We also improved the presentation of data, including statistics reporting and visualization, to meet reproducibility standards. We appreciate the reviewer's constructive suggestions, which have strengthened the clarity and rigor of the manuscript.

This study offers intriguing insights into the antifungal mechanisms of R. nasutus against C. neoformans, particularly regarding virulence inhibition and synergistic potential. However, major revisions are required to ensure the study accurately reflects its scope, demonstrates statistical rigor, and is properly contextualized within existing literature. Addressing these points will significantly strengthen the manuscript's scientific contribution.

Response: We thank the reviewer for the thoughtful and constructive feedback on our manuscript. In response, we have undertaken major revisions to improve the clarity, rigor, and context of the study. Specifically, we have: (i) refined the scope of the manuscript to focus exclusively on *C. neoformans*, with corresponding adjustments to the title and text; (ii) added details of the statistical analyses performed, including sample size, methods, and criteria, now clearly described in the Materials and Methods and indicated in the figure legends; and (iii) expanded the Introduction and Discussion sections to better contextualize our findings within relevant literature on antifungal activity and virulence inhibition.

We believe these revisions have significantly strengthened the manuscript and improved its scientific contribution in line with the reviewer's suggestions.

References

1. Shakya K. 2015. Preparation of Standardized *Rhinacanthus nasutus* Leaf Extract by Green Extraction Methods and Evaluation of Antifungal Activity of Its Topical Solution against Trichophyton rubrum. Prince Songkla University.

2. Tanaporn Phetruen, Salinthip Thongdechsri, Muthita Khongthongdam, Sittiporn Channumsin, Krai Meemon, Sittinan Chanarat. (In press). Effects of simulated microgravity on biological features and virulence of the fungal pathogen *Cryptococcus neoformans*. AEM https://doi.org/10.1128/aem.01435-25.
