# Supplementary material for: Antifungal potential of Rhinacanthus nasutus extracts against the pathogenic fungus Cryptococcus neoformans
Source: PLoS One. 2025 Dec 19;20(12):e0339279. doi: 10.1371/journal.pone.0339279 (PMC12716790; doi:10.1371/journal.pone.0339279)
Supplement: Attachment — Submitted filename: Point-by-point response 251129.docx [file pone.0339279.s009.docx]

**Point-by-point response**

**Reviewer #3:**

*The study aligns well with the journal’s scope, providing novel insights into a plant-based antifungal agent by identifying Rhinacanthus nasutus root extract as a promising candidate for further development. Notably, the authors have thoroughly addressed previous comments and feedback from other reviewers, enhancing the clarity, robustness, and reproducibility of the manuscript. With minor revisions,*

*This manuscript presents an observational study on the antifungal properties of R. nasutus (snake jasmine) and its effects on Cryptococcus neoformans. The authors employ a combination of HPLC quantification, spot assays, and virulence factor analysis to demonstrate that root extracts of R. nasutus significantly inhibit fungal growth and diminish key pathogenic traits, including melanin production, capsule formation, and cell morphology. These findings are supported by clear experimental evidence and are well integrated into the existing literature. Additionally, the near-synergistic interaction with amphotericin B is a significant contribution, suggesting potential therapeutic applications as an adjuvant to current antifungal treatments. Its dual action—growth inhibition and virulence reduction—highlights its potential as both a direct antifungal agent and a synergistic partner with existing drugs.*

***Response***: We sincerely thank the reviewer for the positive evaluation and encouraging remarks. We are pleased that the revisions have improved the clarity and robustness of the manuscript and that the significance of our findings—including the antifungal and virulence-reducing effects of R. nasutus—is well recognized. We appreciate the constructive feedback and have addressed all minor points accordingly.

Minor Revisions:

Figure 1: Graphs (C-E), the Y-axis have different scales, which can mislead readers when comparing peaks and their respective ratios.

***Response***: We thank the reviewer for this helpful suggestion. To facilitate direct comparison, we have generated a new supplementary figure that combines all three graphs into a single plot with a unified Y-axis scale. We chose to keep the original individual panels in the main figure because combining them there reduced readability and risked confusing readers. The new combined plot is now provided as S1 Fig for easy comparison.

Figure 2: To ensure reproducibility, what was the initial concentration of fungal cells used for the spot assays, and how were they serially diluted?

***Response***: We thank the reviewer for pointing out the need for clarification. For the spot assays, fungal cultures were adjusted to an initial OD_600_ of 0.2, and 2.5 µL of each dilution was spotted onto the plates. Serial dilutions were performed at 10-fold intervals. These details have now been added to the Materials and methods section for clarity and reproducibility.

Discussion, line 255; References for cell morphology and its relationship to structural stability and viability should be included to support these claims and observations.

***Response***: We thank the reviewer for this helpful suggestion. We have now added relevant references, including studies in *C. neoformans*, to support the relationship between altered cell morphology, cell-wall integrity, and viability. These citations have been incorporated into the revised Discussion.

Discussion, line 277; Fungal pathogen cell wall properties change with their environment. It should be noted that infection conditions may change the organization of the cell wall via stress pathways, and may change the efficacy of rhinacanthrin-C, further supporting a need for in-vivo studies.

***Response***: We thank the reviewer for this important suggestion. We have revised the Discussion to acknowledge that *C. neoformans* dynamically remodels its cell wall in response to environmental and host-associated stresses, which may alter susceptibility to rhinacanthrin-C or other active components. We have also added relevant references to support this point and further emphasized the need for in vivo studies to evaluate efficacy under infection-related conditions.

**Reviewer #4:**

This is a potential research paper to explore the activity of the extracts from the traditional Thai plant *Rhinacanthus nasutus* against the pathogenic fungus *Cryptococcus neoformans*. The overall framework of the paper is complete, and the research has clear practical significance. However, before moving towards publication, there are several key issues that need to be carefully revised and supplemented by the author.

***Response***: We thank the reviewer for the positive evaluation of our work and for recognizing the practical significance of exploring *Rhinacanthus nasutus* extracts against *Cryptococcus neoformans*. We appreciate the constructive feedback and have carefully addressed all points raised. The manuscript has been revised accordingly to improve clarity, completeness, and scientific rigor.

1.The full text refers to “biological replicates ( n = 2 ) ”. In biological experiments, the number of repetitions of n = 2 is usually considered to be insufficient, and meaningful statistical analysis cannot be performed, and the reliability and variability of experimental results cannot be evaluated. It is recommended to increase the number of biological replicates to at least n = 3.

***Response***: Thank you for the comment. All experiments in this study were performed with at least two independent biological replicates, which showed consistent and reproducible results. While we agree that additional replicates can strengthen statistical power, the variation between our replicates was minimal, and we have been careful not to overstate our conclusions. Given the scope and consistency of the dataset, we believe the current number of biological replicates is sufficient to support the findings presented.

2.Introduction : When the research gap is introduced, the limitations of current antifungal drugs ( especially for *Cryptococcus*), such as the nephrotoxicity of amphotericin B and the resistance of fluconazole, can be more emphasized, thus highlighting the urgency and innovation of this study to explore plant replacement therapy.

***Response***: Thank you for this valuable suggestion. We have revised the Introduction to more clearly highlight the limitations of current antifungal therapies, including amphotericin B–associated nephrotoxicity and increasing fluconazole resistance. This addition strengthens the rationale for exploring plant-derived antifungal agents such as *R. nasutus.*

3.Is the extract concentration in the full text“ mg / mL ”or“mg / mL”( milliliter should be capitalized ) ? Please check it carefully.

***Response***: Thank you for pointing this out. We have carefully checked the entire manuscript and revised all units to use “mL” consistently.

4.The position of rhinacanthin-C should be indicated in Fig.1C-E.

***Response***: Thank you for the suggestion. We have now indicated the putative rhinacanthin-C peak in Fig. 1C–E using an asterisk on each chromatogram.

5.It is still puzzled that the author used HPLC to determine the role of rhinacanthin-C in this paper. Although rhinacanthin-C has the highest content in leaves, roots and root-leaf mixture, it cannot be concluded that it is a substance that inhibits the activity of Cryptococcus neoformans, because a series of experiments on R.nasutus extract were carried out later.

***Response***: Thank you for the comment. Our intention was not to attribute the antifungal activity solely to rhinacanthin-C. To avoid any possible misunderstanding, we have revised the Discussion to clarify that all biological effects were observed using the crude extract, which contains multiple phytochemicals, and that other constituents may also contribute to the antifungal and antivirulence activities.

6.Please revise the reference format to ensure that all species names are in italics, including references, etc., the eighth reference has no name, and all reference titles are in the same case, etc., to confirm whether the journal name is an abbreviation or a full name;

***Response***: Thank you for pointing this out. We have carefully checked and corrected the reference list to ensure consistent formatting, including italicization of species names, completion of missing information, uniform title case, and appropriate journal name formatting according to the journal’s guidelines.

7.Figure S1 should have a statistical analysis, such as counting the size of the point.

***Response***: Thank you for the suggestion. We have now included quantitative analysis of the dot spot sizes, and these results are presented in the revised S2 Figure.

8.Figure 4. Why choose the third day and the fifth day for statistics

***Response***: Thank you for the comment. Our analysis followed the approach used in Fernandes et al. (2022), which measured cell and capsule size on day 5. We also included day 3 in our study to capture earlier morphological dynamics and provide a more complete view of the size changes over time.

Table S1 should be supplemented by KN99α

***Response***: Thank you for the comment. KN99 was already included in the original table with its mating type α indicated in the adjacent column; however, to improve clarity, we have now added the mating type directly next to the strain name as well.

**References:**

1. Fernandes KE, Fraser JA, Carter DA. Lineages Derived from Cryptococcus neoformans Type Strain H99 Support a Link between the Capacity to Be Pleomorphic and Virulence. Bahn Y-S, editor. mBio. 2022;13: e00283-22. doi:10.1128/mbio.00283-22
